# MALDI-MSI: A Powerful Approach to Understand Primary Pancreatic Ductal Adenocarcinoma and Metastases

**DOI:** 10.3390/molecules27154811

**Published:** 2022-07-27

**Authors:** Juliana Pereira Lopes Gonçalves, Christine Bollwein, Anna Melissa Schlitter, Mark Kriegsmann, Anne Jacob, Wilko Weichert, Kristina Schwamborn

**Affiliations:** 1 Institute of Pathology, School of Medicine, Technical University of Munich, Trogerstraße 18, 81675 Munich, Germany; juliana.goncalves@tum.de (J.P.L.G.); christine.bollwein@tum.de (C.B.); melissa.schlitter@tum.de (A.M.S.); anne.jacob@tum.de (A.J.); wilko.weichert@tum.de (W.W.); 2 German Cancer Consortium (DKTK), Partner Site Munich, 81675 Munich, Germany; 3 Institute of Pathology, University of Heidelberg, 69120 Heidelberg, Germany; mark.kriegsmann@med.uni-heidelberg.de

**Keywords:** pancreatic ductal adenocarcinoma, mass spectrometry imaging, metastasis, tumor development, proteomics, prognosis

## Abstract

Cancer-related deaths are very commonly attributed to complications from metastases to neighboring as well as distant organs. Dissociate response in the treatment of pancreatic adenocarcinoma is one of the main causes of low treatment success and low survival rates. This behavior could not be explained by transcriptomics or genomics; however, differences in the composition at the protein level could be observed. We have characterized the proteomic composition of primary pancreatic adenocarcinoma and distant metastasis directly in human tissue samples, utilizing mass spectrometry imaging. The mass spectrometry data was used to train and validate machine learning models that could distinguish both tissue entities with an accuracy above 90%. Model validation on samples from another collection yielded a correct classification of both entities. Tentative identification of the discriminative molecular features showed that collagen fragments (COL1A1, COL1A2, and COL3A1) play a fundamental role in tumor development. From the analysis of the receiver operating characteristic, we could further advance some potential targets, such as histone and histone variations, that could provide a better understanding of tumor development, and consequently, more effective treatments.

## 1. Introduction

**Figure 1:**
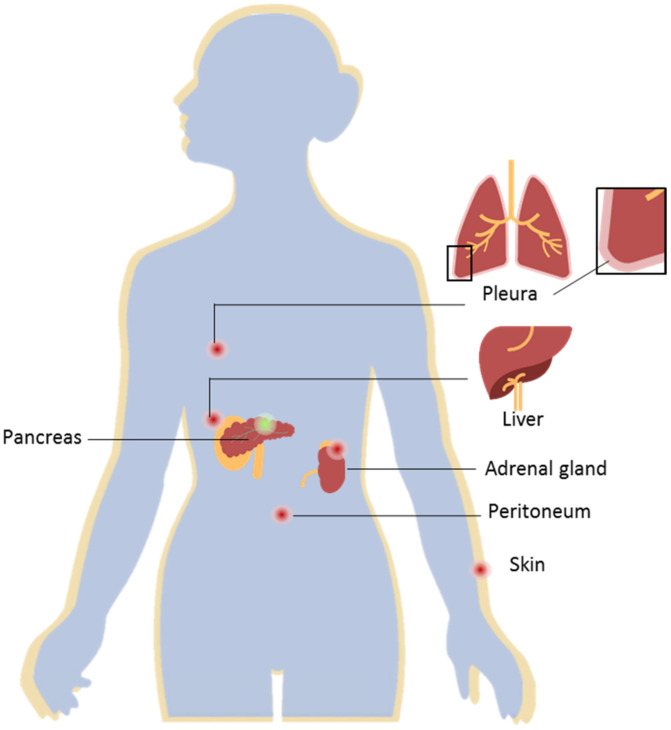
Pancreatic ductal adenocarcinoma (PDAC) and distant metastases. PDAC located in the pancreas (represented by the green dot) presents a high metastatic rate. Distant PDAC metastases are most commonly found in the liver, peritoneum, lungs, adrenal gland, and skin (represented by the red dots). The development of distant metastases is often associated with a poor prognosis for this ailment.

Pancreatic ductal adenocarcinoma (PDAC) constitutes about 90% of pancreatic tumors and remains as one of the most aggressive malignancies, with a 5-year survival rate of 8.5% [1,2]. The poor survival rate of these patients is often related to the rapid development of the disease, with a lack of symptoms in the early stages, and the development of distant metastases. PDAC metastases are usually found in the liver (approximately 80%), lung, and peritoneum (Figure 1) [2,3]. For 80–85% of the PDAC cases with locoregional or distant metastases, surgical treatment cannot be applied; thus, chemotherapy must be administered to improve patients’ survival as well as relieve symptoms. However, current treatment options are limited, they lack specificity, and often produce toxic side effects [4]. Additionally, commonly administered treatment regimens are often focused on the primary tumor, while not efficiently treating the metastasis. This is commonly referred to as mixed or dissociated response to treatment, which means that responding and non-responding lesions coexist in one patient [5]. Currently, little is known about the possible biological explanations or mechanisms that cause this common problem in the treatment of metastatic patients. One possible explanation may be that genomic instability causes genotypic differences between primary tumor and metastasis [6]. Nonetheless, studies have shown that there are no significant differences at the gene or pathway levels in primary and metastatic pancreatic cancers [7]. Another factor that influences mixed response to treatment is the microenvironment, which differs between primary and metastatic lesions [8]. This difference in turn can also influence and change the proteome of cancer cells, a fact that has been proven for many different tumor types, such as breast cancer, colon cancer, and pancreatic neuroendocrine neoplasms [9,10,11]. Recently, it has also been observed in PDAC [7]. Thus, it remains a pressing need to elucidate the changes induced by the divergent microenvironments between primary and metastatic tumors, in order to uncover molecular features for the development of novel treatment options that better address the main causes of mortality and improve patients’ overall survival.

The heterogeneity of this disease has not been successfully characterized with transcriptomics or genomics; however, recent advances in the field of proteomics show auspicious outcomes for the understanding and stratification of this ailment [2,4,12,13,14,15]. 

Mass spectrometry imaging (MSI) enables the characterization of the proteomic content directly on the tissue through a spatially resolved approach. Without requiring any external labeling (e.g., antibodies), MSI enables one to find correlations between a variety of different analytes, such as metabolites, lipids, glycans, or, as used in the present investigation, peptides/proteins, and the histomorphological attributes of the tissue [16,17,18,19]. Furthermore, it is possible to use the same tissue sections for hematoxylin and eosin (H&E), or for immunohistochemical (IHC) staining after MSI analysis; when required, the same tissue is also available for further pathological evaluation [20]. Due to the untargeted nature of this approach, MSI, and in particular, matrix-assisted laser desorption/ionization (MALDI) MSI, allows for the rapid measurement of peptide/protein content, as well as its localization within the tissue. Using this technology, we evaluated the peptide/protein content of tumor samples collected from patients diagnosed with PDAC who also developed metastases. By adopting machine learning tools, we predicted the ability of supervised classification models to differentiate between primary and metastatic tumor tissue. Based solely on the evaluation of tumor content, we identified potential targets for the diagnosis of PDAC metastases, and for the treatment of this disease.

## 2. Results

For an initial approach to explore the differences in primary PDAC and its metastatic lesions, we collected samples from 13 patients. Multiple cores (to increase tissue variability due to the heterogeneous nature of the tissue) from the primary tumors as well as metastases were used to build one mixed tissue microarray (TMA). From five of these patients, it was possible to include matched samples from both primary and distant metastases.

**Figure 2:**
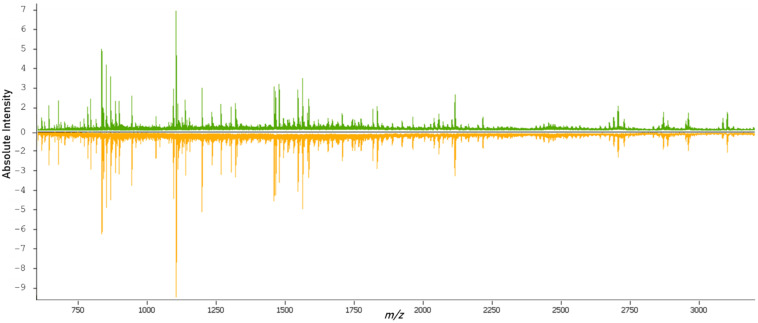
Overview of the average mass spectrum of the primary (green) and metastatic (orange) tissues.

After tryptic digestion, the proteomic content of the samples was measured. From the overview of the spectral data of both classes (Figure 2), it was possible to see variations in the molecular compositions of both classes. The data from the individual spectra were then used for the training and validation of classification models.

### 2.1. Training and Testing of the Classification Models

The spectra generated from annotated tumor regions of the mixed TMA were used to generate different classification models. Patients’ characteristics are summarized in Table 1. 

Three different algorithms were employed to build classification models. 70% of the collected spectra were utilized for training and validation, while 30% were dedicated to test the model. The overall accuracies obtained are summarized in Table 2, as well as sensitivity and specificity of the classification models, as calculated by the confusion matrices (Table S1).

### 2.2. External Validation of the Classification Models

The classification models were also tested on an external validation data set (*n* = 25) obtained from samples that were collected at a different clinical site. For the classification of this test set, the applied classification models performed similarly, with classification accuracies obtained between 89.5% and 93.7%. RF and LDA were the most sensitive methods, while higher specificity was achieved when applying SVM.

**Figure 3:**
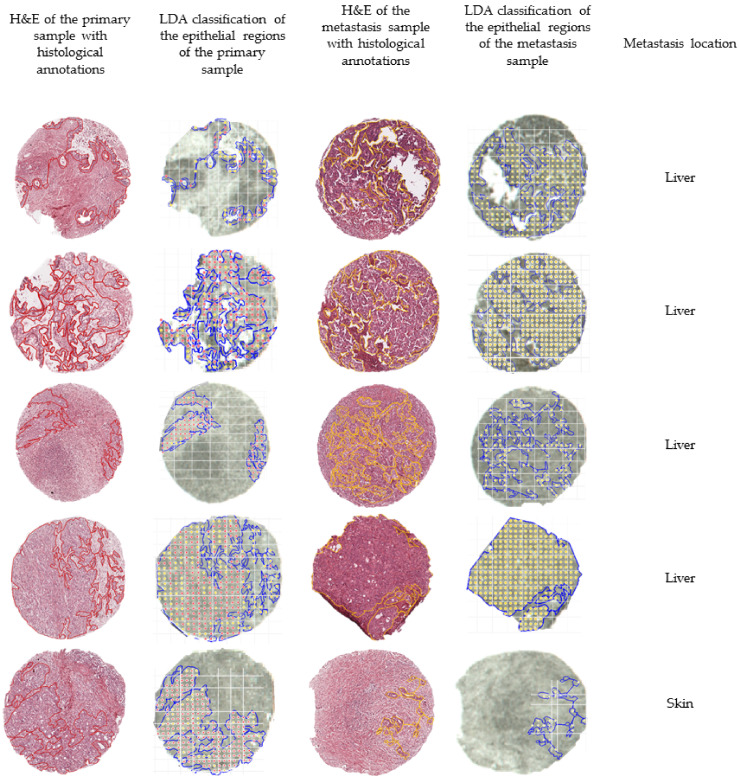
Overview of the classification outcomes obtained for the external validation data set. The annotated regions of the primary (in red) and the annotated regions of the metastases (in yellow) were subjected to the built classification models. The classification outcomes that used the linear discriminant analysis (LDA) model were plotted and overlaid with the measurement regions. Classifications of primary tumors are represented with red dots, while classifications of distant metastases are represented by yellow dots.

In order to further evaluate the external validation, we mapped the outcome of the classification of five matched pairs of primary and metastatic tumors, meaning that the samples were collected from the same patient. Figure 3 summarizes the outcomes of the external validation using the LDA model.

### 2.3. Feature Selection

The *m*/*z* values with the highest-intensity differences between primary and metastatic tumors are summarized in Table 3. The *m*/*z* values with the highest impact for LDA classification are summarized in Table S2, as calculated by forward feature extraction. By interpretation of these results, we can see that collagens, namely collagen type I α-1 (COL1A1), collagen type I α-2 (COL1A2), and collagen type III α-1 (COL3A1), are the features with the highest impact in the classification model. Additionally, from the area under the curve of the receiver operating characteristic (AUC-ROC) summarized in Table 3, core histone macro-H2A.1 (MACROH2A1), histone PARylation factor 1 (HPF1), collagen type IV α-3 (COL4A3), tubulin beta-2C (TBB2C), actin, and histone H2B (H2B) are overexpressed in the metastatic lesions.

## 3. Discussion

While the etiology of PDAC can be better understood through investigation of the primary lesion, characterization of the metastases can uncover the molecular features for the development of novel treatment options that better address the main cause of mortality and improve the patients’ survival overall. Proteomics can disclose the real activities of the targets in tumors, as well as in the corresponding metastases, by exhibiting individual molecular profiles from the different tissue lesions and consequently offering explanations for dissociated or mixed treatment responses.

In this preliminary study, we evaluated the capability of mass spectrometry imaging, namely MALDI-TOF-MSI, to be used as a tool to further elucidate the peptide/protein differences between primary PDAC and its distant metastasis. Following sample preparation and measurement, the obtained mass spectrometry data were used to train, test, and validate supervised classification methods. For training and testing the method, a mixed TMA with samples from both primary PDACs and distant metastases was utilized. From each sample, multiple cores from distinct areas of the tumor tissue were included in the TMA, with the intent of including as much tumor heterogeneity as possible. In this TMA, a total of 30 cores with significant tumor content from 13 patients were measured. Moreover, matched samples paired from both the primary and metastatic tumors of five patients were also included, in order to decrease the number of inter-personal deviations in the data set. Additionally, by considering a mixed TMA in building the classification model, we eliminated any inter-measurement bias.

Further matched pairs were collected at a different pathology laboratory (University Hospital of Heidelberg (*Universitäts Klinikum* Heidelberg, UKHD)), and herein employed as external validation of the built models. These samples were also measured by MALDI-TOF-MSI, and the obtained spectrometry data were subjected to classification using the built models. The external validations of primary and metastatic PDAC pairs of five patients were mapped to better assess the efficiency of the best performing model—LDA.

As previously mentioned, the tryptic peptide content of annotated tumor regions of the mixed TMA, measured by MALDI-TOF, was used for the training and testing of three supervised classification models, which yielded good accuracies (approximately 90% for all employed models). The classification models were then applied to the samples from UKHD for further validation of the model, by classifying the annotated tumor areas of matched pairs of tissue samples from patients previously diagnosed with PDAC who had also developed distant metastases. Most machine learning models are trained and tested on the same data set that often is built from samples from the same institution; this approach can introduce a systematic bias. Hence, this validation step is important in assessing the possibility of applying the machine learning models, built from a dataset collected at one site, to classify samples collected from different institutions. As each institution has an internal operation procedure, using different devices and employing different solvents, the samples used for the external validation were subjected to a slightly different fixation procedure, which introduces some sample preparation variability. Nonetheless, the built models were capable of accurately classifying both primary and metastatic tumors, further highlighting the robustness of the approach. Notwithstanding, some areas of the primary samples were misclassified, which is likely a reflection of an imperfect co-registration process. PDAC samples often present high cellular heterogeneity; therefore, the histological annotation of epithelial regions is a meticulous process that results in smaller regions of interest. The co-registration between the histological annotations and the measurement regions of the validation data set was carried out on a core-by-core basis in order to minimize possible co-registration issues; however, it cannot be disregarded that an overlay error could have occurred, resulting in a less successful classification outcome for the primary tissue. A similar effect could also have occurred if the sample teaching (preceding the measurement) was not accurately matched. Moreover, a laser size of 50 µm can also be a limitation, as this measurement width can be too large to accommodate smaller regions in the tissue; consequently, possible “contamination” of the epithelial tumor region with stromal or inflammatory cells could have occurred during the measurement. Furthermore, there is also an imbalance in the spectra of the epithelial regions of each class in the mixed TMA. Of the samples, 70% of them are from distant metastases, while only 30% of the spectra are from PDAC primary tumors. Additionally, the features that play a more pronounced effect in the classification, as obtained by feature extraction, were found to be overexpressed in the metastases. This translates into a better-defined classification profile of the metastatic tissue. From the external validation, it was also found that larger regions of tumor were better classified in comparison with small tumor regions (Figure S1). The reasoning behind this effect is once again explained by the co-registration effects and laser measurement size.

Another consideration about this study is that most of the metastasis samples utilized to build the classification models were collected from the liver (70%), and the remaining were collected from the peritoneum. Despite being close approximations of the real values, there are likely some *m*/*z* features that are affected by this imbalance. This might also impact the overall accuracy of the classification, especially if the tested tissue type was not included in the initial training of the method (e.g., skin and adrenal gland).

From the analysis of the most relevant *m*/*z* features for the classification, as calculated by forward feature extraction, we can conclude that COL1A1, COL1A2, and COL3A1 play a central role in tumor progression. As also indicated by other studies, collagen content can impact the progression of tumors with different origins [26,27,28,29,30,31,32,33,34], which can likely be attributed to the role of collagen in cellular migration and tumor progression [35,36]. Moreover, in PDAC, collagens have been associated with poor prognoses [37,38,39,40].

A study from Loch et al., where MALDI-MSI was used to investigate prognostic markers of lymphatic vessel invasion, lymph node metastasis, and angioinvasion in pancreatic cancer, it was determined that actin, COL2A1, COL4A3, filamin-B, histone H1.3, spectrin β-chain, non-erythrocytic, vasolin-containing protein, and vinculin are peptide signatures of lymphatic vessel invasion; COL2A1 and myosin-11 were identified as prognostic signatures of angioinvasion; and histone 1.3 was associated with lymph node metastases [25]. These results portray the pivotal role of collagens in the progression of pancreatic malignancies. These findings also agree with what was investigated by Tian and co-workers. In their report, the authors advance that differential responses to fibrillar collagens derived from epithelial or stromal cells may provide another way of stratifying patients—high vs. low levels of COL1A1 expressed by epithelial cells—which may also lead to differing responses to treatments that alter fibrillar collagen expression [40]. Further understanding of the role of collagens in the development of PDAC could potentially be achieved by MSI analysis by employing a dedicated enzymatic digestion.

From the analysis of the features that differ the most between both classes through extracting the features with the highest receiver operation characteristic (ROC), we were able to identify MACROH2A1, HPF1, COL4A3, TBB2C, actin, and H2B as being overexpressed in the metastatic lesions in comparison with their primary tumors. Histones bring genetic information into the nuclear space, and contribute to the regulation of all DNA template-based reactions. In human somatic cells, there are eight variants of H2A and six variants of H3 [41]. Additionally, testis-specific histone H2B has also been identified. The different histone variants have been correlated to specific transcription functions, possessing also different protein sequences and roles in post-translational modifications. Therefore, deregulation of histone variants plays a pivotal role in tumor origin and progression. The tentative identification of three peptide fragments showed that core histone macro-H2.1 (MACROH2A1), histone PARylation factor 1 (HPF1), and histone H2B are overexpressed in PDAC metastases when directly compared to their primary tumors. Over the past decade, attention has been brought to the involvement of MACROH2A in cancer biology, and especially in the proliferation of metastases [42,43,44,45]. The epithelial–mesenchymal transition (EMT) is important during the proliferation of epithelial cancers, and here MACROH2A1 has significant structural similarity to the canonical histone H2A, differing in size since it has an evolutionarily conserved non-histone macro domain of ~25 kDa. [46,47]. This protein, similarly to H2A, plays a central role in transcription regulation as well as DNA replication and repair [41,48]. The protein HPF1 is essential for the mediation of the transPARylation of histones, while also switching the specificity of PARylation from Asp/Glu to Ser residues [49].

The tentative identification of three peptide sequences that relate to histone variants and products shows a clear involvement of the histone and histone-derived proteins in the cellular proliferation of PDAC distant metastases; however, additional investigation needs to be conducted to further understand the role and correlation of the three protein entities in the progression of the disease.

COL4A3 belongs to the type IV collagen family, which is the main component of basement membranes (BMs) that surround blood vessels. For malignant cells to metastasize they have to penetrate through the epithelial basement membrane (EBM) as well as the BM that surrounds blood vessels. Overexpression of COL4A3 has been correlated with tumor size, higher grade, metastasis, and invasion in several malignancies [50,51,52,53,54,55,56].

TBB2C, also known as tubulin 4B chain, belongs to class IVB β-tubulin, which heterodimerizes with α-tubulins to form microtubules during the transition from metaphase to anaphase of mitotic cellular division [57]. An alteration in the isotopic composition of the tubulin can result in a different dynamic of microtubule formation, which in turn can lead to an alteration in the tumor-budding grade and invasion of cancer cells, leading to proliferation of the tumor cells [58]. Up-regulation of TBB2C has been reported in sentinel lymph node metastases of colorectal cancer and ovarian cancer [57,58].

While the involvement of the identified proteins for the development of PDAC requires further investigation, it is clear from the existing literature that they facilitate cellular proliferation and tumor progression.

No further consideration of actin as a possible marker was given. As actin is highly abundant in the epithelial tissue, only very precise and sensitive methodologies could be used for its precise quantification and validation, which would insignificantly impact diagnostic capabilities.

Selected features from the AUC-ROC analysis of the mixed TMA were also evaluated in the external validation data set. Figures S2 and S3 show a comparison of the intensity of the *m*/*z* features 1198.7 (actin), 1220.6 (COL4A3), 1493.7 (COL1A2), and 1039.5 (tubulin beta-2C chain) in both data sets. These features showed comparable expression behaviors between both data sets, underlying the discriminative role of these proteins.

In order to develop novel treatment courses, it is imperative to improve the insight into disease development and assist with identification of specific therapeutic targets; these measures will subsequently enable a more efficient, targeted therapy. Novel treatment courses are also necessary for lesions that frequently present dissociate responses, such as metastatic PDAC, where the use of different treatment targets will likely improve treatment outcomes.

## 4. Materials and Methods

### 4.1. Sample Collection

The patient cohort of the mixed TMA was assembled by searching the clinical database of the Institute of Pathology from the Technical University of Munich, in order to identify cases of PDAC that had also developed metastases. Pairs of samples (primary tumor and metastasis from the same patient) from patients with surgical treatment between 2008–2019 were included in the study. The patient cohort of two additional TMAs (primary tumor–TMA P and metastasis–TMA M) was obtained by searching the database of the Pathology Institute of the University Hospital of Heidelberg, in order to identify patients that had been diagnosed with PDAC who had also developed distant metastases. Pairs of samples (primary tumor and metastasis from the same patient) from patients with surgical treatment between 2008–2014 were included in the study.

Standard protocol of histopathological work-up of resected specimens was performed for conventional pathological evaluation and risk stratification of tumors. The patient data were obtained from the internal clinical data repository of their respective clinical sites. This study was approved by the local institutional review board and performed according to the national rules (Approval 403/17S). The study was carried out in compliance with the Helsinki Declaration.

### 4.2. TMA Construction

#### 4.2.1. Mixed TMA

In order to allow for high-throughput analyses, selected areas of the tumor tissue from each patient (*n* = 13) were combined into one TMA. Tumor tissue used for TMA construction was initially fixed in 4% buffered formalin and embedded into paraffin. Patient samples were randomly distributed across the TMA. Replicate tumor cores from each patient were placed, one after another. The TMA contained two, three, or four cores (1.0 mm in diameter) from each patient. The TMAs were produced with the Tissue Arrayer (Alpha Metrix Biotech GmbH, Rödermark, Germany). After completing a TMA block, it was heated at 38 °C for 60 min.

#### 4.2.2. Primary and Metastasis Validation TMAs (TMA P and TMA M)

Tumor samples from patients previously diagnosed with PDAC that developed metastases (*n* = 26) were assembled into two different TMAs. One TMA contained the primary tumors, while the second TMA contained the metastatic lesions. All samples were included in duplicates, with the exception of one patient from whom more samples were included. The TMAs were constructed as described in [17,59].

### 4.3. MALDI-MSI Measurement

From each TMA, a 4-micrometer section was adhered to an indium-tin-oxide (ITO) coated glass slide (Bruker Daltonics, Bremen, Germany). Sample preparation has previously been described in detail [60]. Briefly, sample slides were heated to 80 °C prior to dewaxing with xylene (Carl Roth GmbH, Karlsruhe, Germany), and subsequently rehydrated with graded ethanol washes (Carl Roth GmbH). Afterward, samples were subjected to heat-induced antigen retrieval in MilliQ water at 95 °C for 20 min. A trypsin (Promega, Mannheim, Germany) solution was prepared in 40-millimolar ammonium bicarbonate (Sigma-Aldrich Chemie GmbH, Munich, Germany) to a final concentration of 0.1 µg/µL for on-tissue digestion. The trypsin solution was sprayed, utilizing an automatic sprayer (TM Sprayer, HTX Technologies, Chapel Hill, NC, USA) in 16 cycles with a fixed spraying flow rate of 150 μL/min (distribution of 5 × 10^−3^ µg/mm^2^). The samples were digested in a humidity chamber at 37 °C for two hours. Subsequently, four cycles of matrix solution, 10 mg/mL of α-cyano-4-hydroxycinnamic acid matrix (Sigma-Aldrich Chemie GmbH) in 70% acetonitrile aqueous solution with 1% trifluoracetic acid (Carl Roth GmbH), were sprayed at a flow rate of 120 μL/min (deposition of 2 × 10^−3^ µg/mm^2^).

MSI was performed using a rapifleX^®^ MALDI Tissuetyper^®^ TOF mass spectrometer-rapifleX MALDI - time-of-flight (TOF) mass spectrometer (Bruker Daltonics). A peptide calibration standard mix (bradykinin, angiotensin II, angiotensin I, substance P, bombesin, ACTH clip 1-17, ACTH clip 18-39, and somatostatin 28 (Bruker Daltonics)) was used for external mass calibration. Each spectrum was automatically generated at a spatial resolution of 50 µm using flexControl (Bruker Daltonics) in the mass range of *m*/*z* 600–3200. A total of 500 laser shots were acquired for each spectrum at 10 kHz, using a laser power of 95%. Laser application was defined as M5 small, and beam scan was turned on with a sample rate of 1.25 GS/s. Real-time smoothing was turned off, while matrix suppression was deflected up to *m*/*z* 479. The global offset attenuator was set at 2%. The measurement regions were defined using flexImaging (Bruker Daltonics). Following MSI measurements, the matrix was removed using two washes in 99.99% methanol (Carl Roth GmbH) for 3 min each, followed by two washings in 99.99% ethanol (Carl Roth GmbH) for 10 s.

### 4.4. Histological Tumor Annotation, Data Processing, and Extraction

Following matrix removal, the same TMA sections that were previously measured by MSI were stained with hematoxylin and eosin (H&E) and digitalized, utilizing a slide scanner (Aperio CS2, Leica Bio-systems, Wetzlar, Germany). H&E scans were uploaded, and tumor regions were thoroughly annotated using SCiLS Cloud (discontinued service from Bruker Daltonics) or QPath (v0.2.2) by a board-certified pathologist [61]. MSI data were processed using SCiLS Lab Pro (Bruker Daltonics) for mass spectrometry and image visualization. Annotations were imported into SCiLS Lab Pro software. The spectra baseline was corrected to the total ion count (TIC).

### 4.5. MS/MS Measurements

Tentative identification of the *m*/*z* features was carried out using MS/MS measurement on whole-mount PDAC tissue sections, utilizing a timsTOFflex mass spectrometer (Bruker Daltonics). Laser power for fragmentation was set at 70% on positive ionization mode for a laser frequency of 5 kHz, and a beam scan of 25 µm^2^. For the identification, the MS/MS spectra (Figures S4–S9) were submitted to MASCOT MS/MS Ion Search, where the SwissProt database was searched to match tryptic peptide sequences with their respective intact proteins, defining *homo sapiens* as the taxonomic class. The MS/MS spectrum search parameters included a mass tolerance of 1 Da, MS/MS tolerance of ±1 Da, up to two missed cleavages, methionine oxidation, protein *N*-terminal acetylation, as well as proline oxidation as variable modifications.

### 4.6. Statistical Analysis

As mentioned in Section 4.4, following the MALDI-TOF-MSI measurements, the matrix was removed from the sample slides and stained with H&E and digitalized; the epithelial regions were carefully annotated by a pathologist. Hence, only tumor regions were taken into consideration for statistical analysis.

#### Supervised Classification

All spectra were preprocessed for intensity profile normalization, resampling, spatial de-noising, and calculation of a second normalization profile, as previously described [62,63]. Summarily, all spectra were recalibrated to the peptide chemical noise background as previously described [62]. Peptide mass resampling was performed in every data analysis, where all spectra were resampled to the expected *m*/*z* values according to the peptide mass rule [63]. For the total ion count (TIC) normalization, all spectra were divided by the sum of their respective intensities. Intensity profile normalization was carried out, where the intensities of the spectra were converted in accordance with a reference distribution profile [63]. The peak picking was performed on the global skyline spectrum in SCiLS Lab, using a peak width of 0.4 Da and peak aggregation mode “sum”. The 431 most intense features in the measured *m/z* range were selected. Afterward, spectra of individual spots were exported to .csv-format files and imported into R statistical software (version 3.6.3) and RStudio 1.2.5033 [64].

The data set was split into training (70%) and test (30%) sets, with method control set to 10-fold cross-validation for all models. Classification models were fitted using the “caret” package on R [28]. Linear discriminant analysis (LDA) was fitted using the method “lda”. Random forest (RF) was fitted using the method “ranger”, with the number of trees set to 50. Tuning parameter ‘min.node.size’ was held constant at a value of 1. Accuracy was used to select the optimal model using the largest value. The final values used for the model were mtry = 432, splitrule = extratrees, and min.node.size = 1. K-nearest neighbors (kNN) was fitted using the method “knn”. Accuracy was used to select the optimal model using the largest value. The final value used for the model was k = 5. Support vector machine (SVM) was fitted using the method “svmLinear3”. Accuracy was used to select the optimal model using the largest value. The final values used for the model were cost = 0.25 and loss = 1.

The fitted models were used to predict the test data subset. The accuracy, sensitivity, and specificity (Table 2) were calculated based on the results of the confusion matrix (Table S1). The same models were also applied on the MSI data recorded of TMA P and TMA M, as an additional validation step to predict their respective classes.

## 5. Conclusions

Pancreatic ductal adenocarcinoma has a very poor prognosis, with an overall 5-year survival rate of less than 10%. Even after successful resection, the survival rate is solely 21%. These somber perspectives are often related to the development of distant metastases and tumor progression. With current treatments not always being efficient in treating the primary tumor and its metastasis simultaneously, there is an eminent need for an approach that targets both the primary lesion as well as its metastasis. From our proteomics methodology, based on direct tissue characterization, we devised three machine learning models that could classify the tissue sections with an accuracy of over 90%. External validation of the models yielded also comparable results, validating the robustness of the approach. From feature analysis we concluded that collagens, MACROH2A1, HPF1, COL4A3, TBB2C, actin, and H2B are promising targets for the understanding of tumor progression and development of distant metastases, and consequently inspiring novel treatment of PDAC metastatic lesions.

## Figures and Tables

**Table 1:** Patients’ characteristics of the mixed TMA.

Patients	*n* = 13
Age (average ± deviation, years)	68 ± 26
Gender	
Female	6
Male	7
T stage	
N/A	4
pT3	8
pT4	1
Nodal involvement	
N/A	4
pN 1	8
pN 2	1
CTx	5
Tumor grading	
N/A	4
2	4
3	5
Metastasis location	
Liver	9
Peritoneum	4

N/A—not available; CTx—neoadjuvant chemotherapy treatment.

**Table 2:** Classification accuracy of the supervised classification models.

Classification Outcome	RF	SVM	LDA
Accuracy	0.9319	0.9368	0.8972
Sensitivity	0.9783	0.9567	0.9783
Specificity	0.8208	0.8892	0.7028

RF—random forest; SVM—support vector machine; LDA—linear discriminant analysis.

**Table 3:** Top 10 features, calculated by AUC-ROC.

*m*/*z*	AUC-ROC	Tentative ID	Sequence	Modifications	MASCOT Score
1336.639	0.66484	Core histone macro-H2A.1	LEAIITPPPAKK	Acetyl (N-term) Oxidation (P)	37
1198.711	0.66414	Actin *	AVFPSIVGRPR		73
976.426	0.65948	Actin	AGFAGDDAPR		^#^ [21,22]
632.313	0.65927	Histone PARylation factor 1	VGGGGKR		31
1220.666	0.65995	Collagen alpha-3 (VI) chain	LGAPGTPGLPGPR	2 Oxidation (P)	40
1493.741	0.64999	Collagen alpha-2(1) chain precursor	GLHGEFGLPGPAGPR	2 Oxidation (P)	40
1039.518	0.6448	Tubulin beta-2C chain			^#^ [23,24]
805.419	0.6441	Collagen alpha-3 (VI) chain			^#^ [25]
901.485	0.6415	Histone H2B	LAHYNKR		43
911.438	0.6391				

* Possible underlying isoforms: ACTA1 or ACTA2, ACTAB, ACTG1, ACTAG2, POTEI, POTEKP, POTEF or POTEE. ^#^ Tentative identification based on literature search.

## Data Availability

The data presented in this study are available on request from the corresponding author.

## Supplementary Materials

The following are available online at https://www.mdpi.com/article/10.3390/molecules27154811/s1, Table S1: Classification confusion matrices; Table S2: Top 10 features, calculated by forward feature selection; Figure S1: PDAC primary samples—the effect of the epithelial region size; Figure S2: Feature expression comparison between primary tissue and metastasis in the mixed TMA; Figure S3: Feature expression comparison between primary tissue and metastasis in the validation data set; Figure S4: MS/MS fragmentation spectra utilized for the tentative identification of *m*/*z* = 632.3; Figure S5: MS/MS fragmentation spectra utilized for the tentative identification of *m*/*z* = 900.5; Figure S6: MS/MS fragmentation spectra utilized for the tentative identification of *m*/*z* = 1335.7; Figure S7: MS/MS fragmentation spectra utilized for the tentative identification of *m*/*z* = 1220.7; Figure S8: MS/MS fragmentation spectra utilized for the tentative identification of *m*/*z* = 1198.7; Figure S9: MS/MS fragmentation spectra utilized for the tentative identification of *m*/*z* = 1493.7.

## Sample Availability

Raw mass spectrometry imaging data used in this report can be made available upon request from the corresponding author.
