# Supplementary material for: MALDI-MSI: A Powerful Approach to Understand Primary Pancreatic Ductal Adenocarcinoma and Metastases
Source: Molecules. 2022 Jul 27;27(15):4811. doi: 10.3390/molecules27154811 (PMC9369872; doi:10.3390/molecules27154811)
Supplement: Supplementary file 1 [file molecules-27-04811-s001.zip › molecules-1825700-supplementary.pdf]

molecules

*Supplementary Materials*

Article

# MALDI-MSI: A powerful approach to understand Primary Pancreatic Ductal Adenocarcinoma and Metastases

# MALDI-MSI: A Powerful Approach to Understand Primary Pancreatic Ductal Adenocarcinoma and Metastases

**Juliana Pereira Lopes Gonçalves [1], Christine Bollwein [1], Anna Melissa Schlitter [1,2], Mark Kriegsmann [3], Anne Jacob [1], Wilko Weichert [1,2] and Kristina Schwamborn [1,*]**

[1] Institute of Pathology, School of Medicine, Technical University of Munich, Trogerstraße 18, 81675 Munich, Germany; juliana.goncalves@tum.de (J.P.L.G.); christine.bollwein@tum.de (C.B.); melissa.schlitter@tum.de (A.M.S.); anne.jacob@tum.de (A.J.); wilko.weichert@tum.de (W.W.)
[2] German Cancer Consortium (DKTK), Partner Site Munich, 81675 Munich, Germany
[3] Institute of Pathology, University of Heidelberg, 69120 Heidelberg, Germany; mark.kriegsmann@med.uni-heidelberg.de
* Correspondence: kschwamborn@tum.de

**Table S1: Classification confusion matrices.** ................................ Error! Bookmark not defined.

**Table S2: Top 10 features, calculated by forward feature selection.** .......................................... 2

**Figure S1: PDAC primary samples - the effect of the epithelial region size..** .......................... 3

**Figure S2: Feature expression comparison between primary tissue and metastasis in the mixed TMA..** Error! Bookmark not defined.

**Figure S3: Feature expression comparison between primary tissue and metastasis in the validation dataset.** . ............................................................................................................................................ 5

**Figure S4. MS/MS fragmentation spectra utilized for the tentative identification of *m/z* = 632.3.**........ 6

**Figure S5. MS/MS fragmentation spectra utilized for the tentative identification of *m/z* = 632.3.**........ 6

**Figure S6. MS/MS fragmentation spectra utilized for the tentative identification of *m/z* = 632.3.** ....... 7

**Figure S7. MS/MS fragmentation spectra utilized for the tentative identification of *m/z* = 1220.7.**...... 7

**Figure S8. MS/MS fragmentation spectra utilized for the tentative identification of *m/z* = 1493.7.**...... 8

**Table S1**. Classification confusion matrices of the three algorithms trained with human PDAC primary and metastasis samples.

| Confusion Matrices | RF | SVM | LDA |
|---|---|---|---|
| Accuracy | 0.9319 | 0.9368 | 0.8972 |
| 95% CI | 0.9177, 0.9444 | 0.923, 0.9488 | 0.8804, 0.9124 |
| p-Value (Acc > NIR) | $< 2.2 \times 10^{-16}$ | $< 2.2 \times 10^{-16}$ | $< 2.2 \times 10^{-16}$ |
| Kappa | 0.8299 | 0.8476 | 0.7336 |
| Mcnemar's Test p-Value | $8.61\times 10^{-8}$ | 0.8339 | $< 2.2\times 10^{-16}$ |
| Sensitivity | 0.9783 | 0.9567 | 0.9783 |
| Specificity | 0.8208 | 0.8892 | 0.7028 |
| Pos Pred Value | 0.929 | 0.9539 | 0.8875 |
| Neg Pred Value | 0.9405 | 0.8955 | 0.9313 |
| Balanced accuracy | 0.8996 | 0.9229 | 0.8406 |

Acc – accuracy; NIR – no information rate; Pos Pred Value – positive predictive value; Neg Pred Value – negative predictive value

**Table S2**. Top 10 features, calculated by forward feature selection

| ***m/z*** | **Accuracy** | **Possible ID** |
|---|---|---|
| 1198.71092 | 0.6237238 | actin |
| 2960.40253 | 0.6340433 | Collagen alpha-2(1) chain precursor |
| 3101.50179 | 0.6577561 | |
| 795.36804 | 0.6805357 | Collagen alpha-1(III) chain |
| 628.31276 | 0.7010649 | |
| 2705.33098 | 0.7119333 | Collagen alpha-1(1) chain precursor |
| 1832.87696 | 0.7215391 | |
| 1478.7627 | 0.7339993 | Collagen alpha-2(1) chain precursor |
| 898.460275 | 0.7460753 | Collagen alpha-1(1) chain precursor |
| 1139.57803 | 0.7512899 | Collagen alpha-1(III) chain |

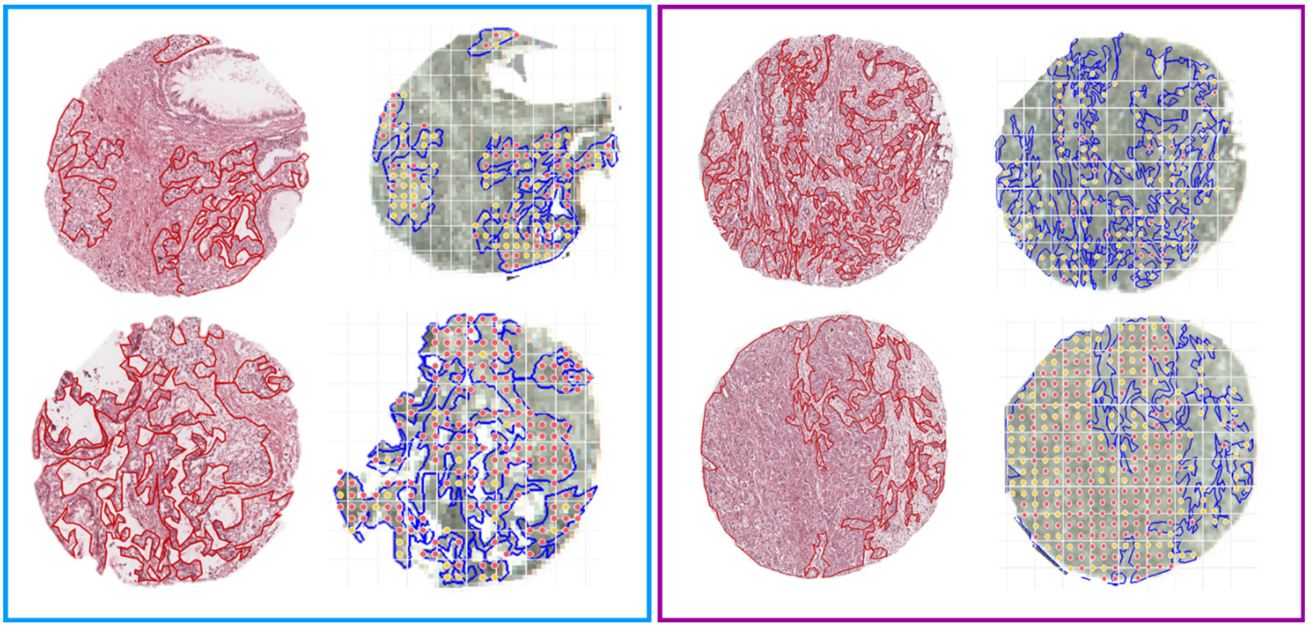

**Figure S1.** PDAC primary samples - the effect of the epithelial region size in the classification outcome. Two different regions from the primary PDAC tumor of the same patient are presented within the blue box and also in the purple box. On the top regions, which are more heterogeneous, it is possible to see that the classification outcome is not as accurate as in the bottom regions, where bigger areas of tumor (epithelial) regions are found. This effect might be do to an inefficient teaching, a defficient co-registration process and/ or the measurement area per pixel not be adequate for the analysis of such detailed regions.

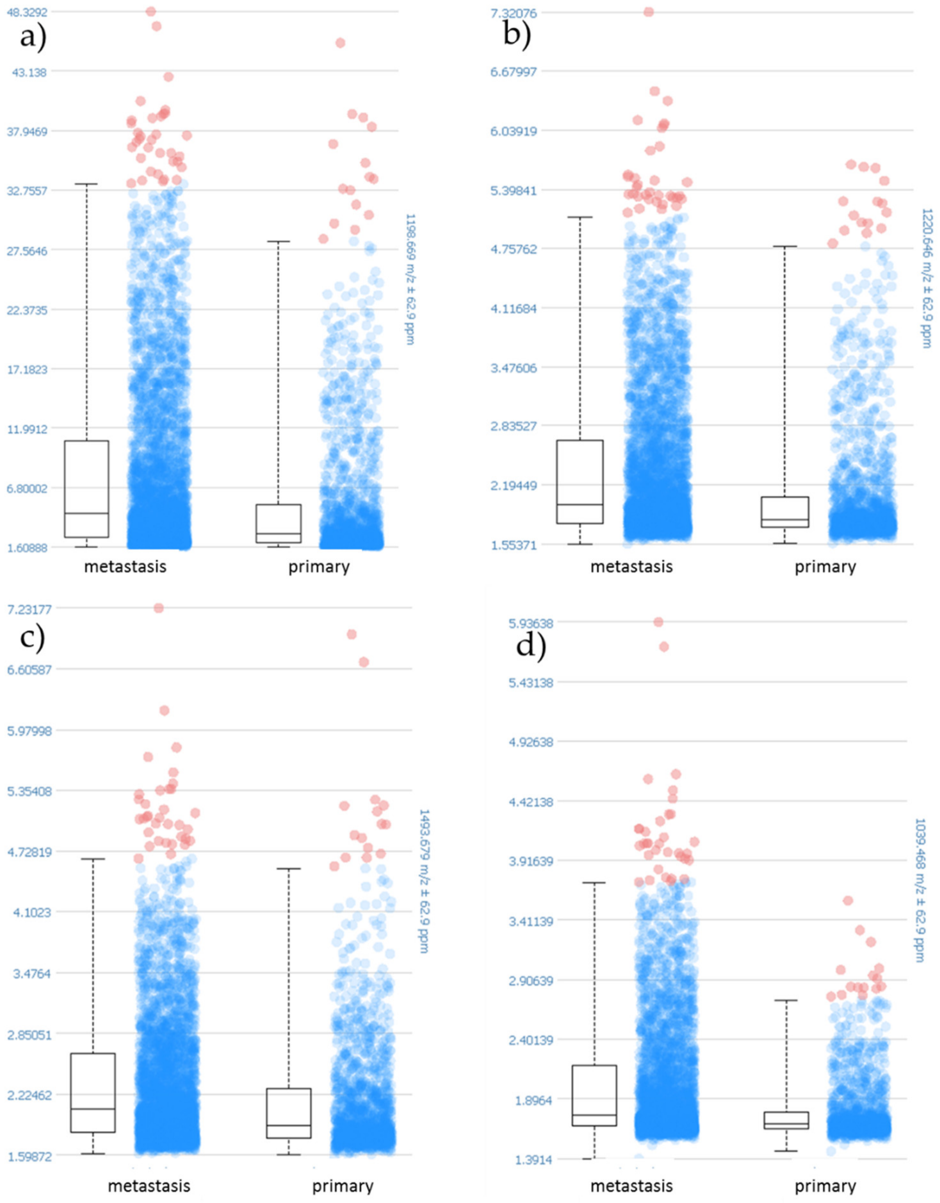


**Figure S2.** Feature expression comparison between primary tissue and metastasis in the mixed TMA. (a) *m*/*z* = 1198.7 (actin), (b) *m*/*z* = 1220.6 (COL4A3), (c) *m*/*z* = 1493.7 (COL1A2), and (d) *m*/*z* = 1039.5 (TBB2C).

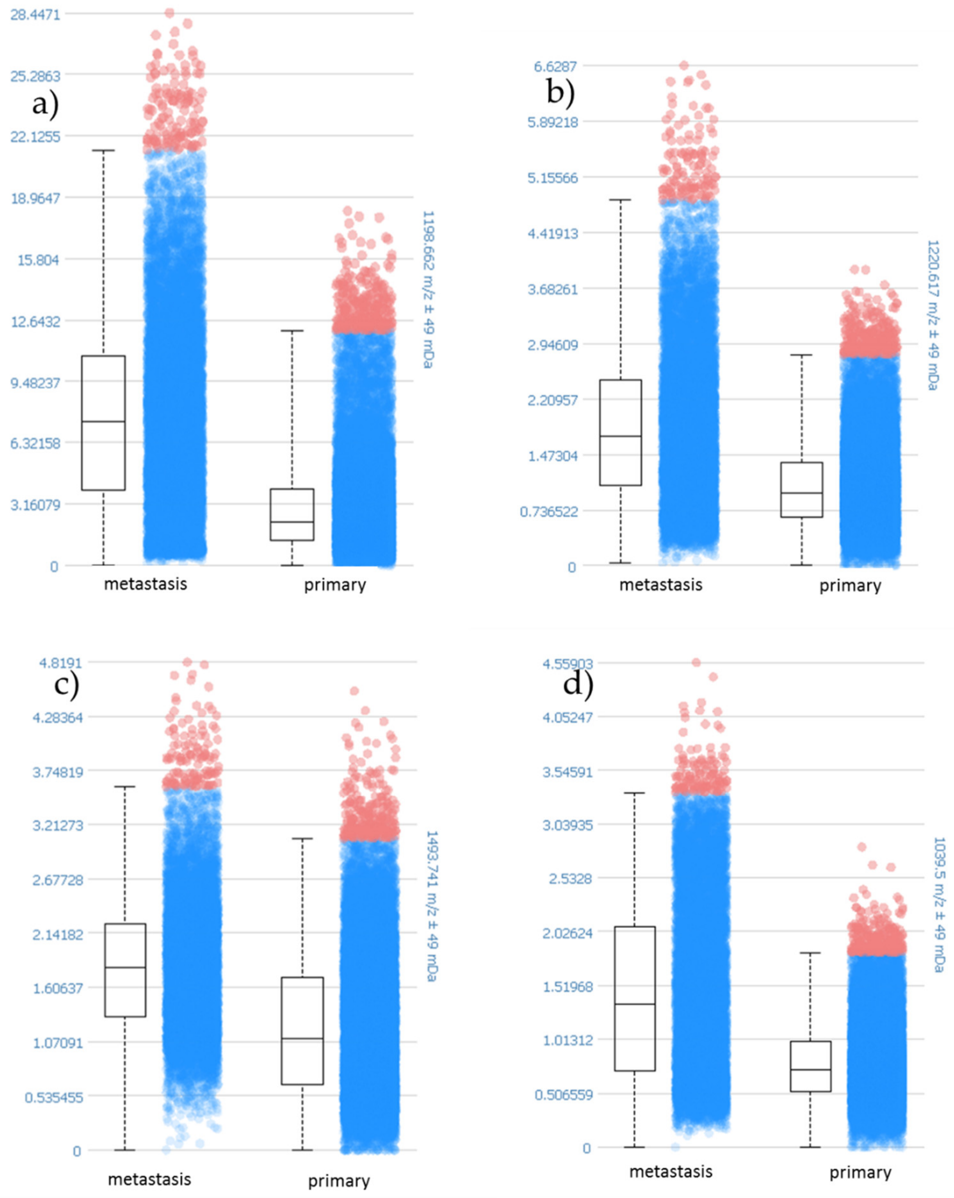


**Figure S3.** Feature expression comparison between primary tissue and metastasis in the validation dataset. (a) m/z = 1198.7 (actin), (b) m/z = 1220.6 (COL4A3), (c) m/z = 1493.7 (COL1A2), and (d) m/z = 1039.5 (TBB2C).

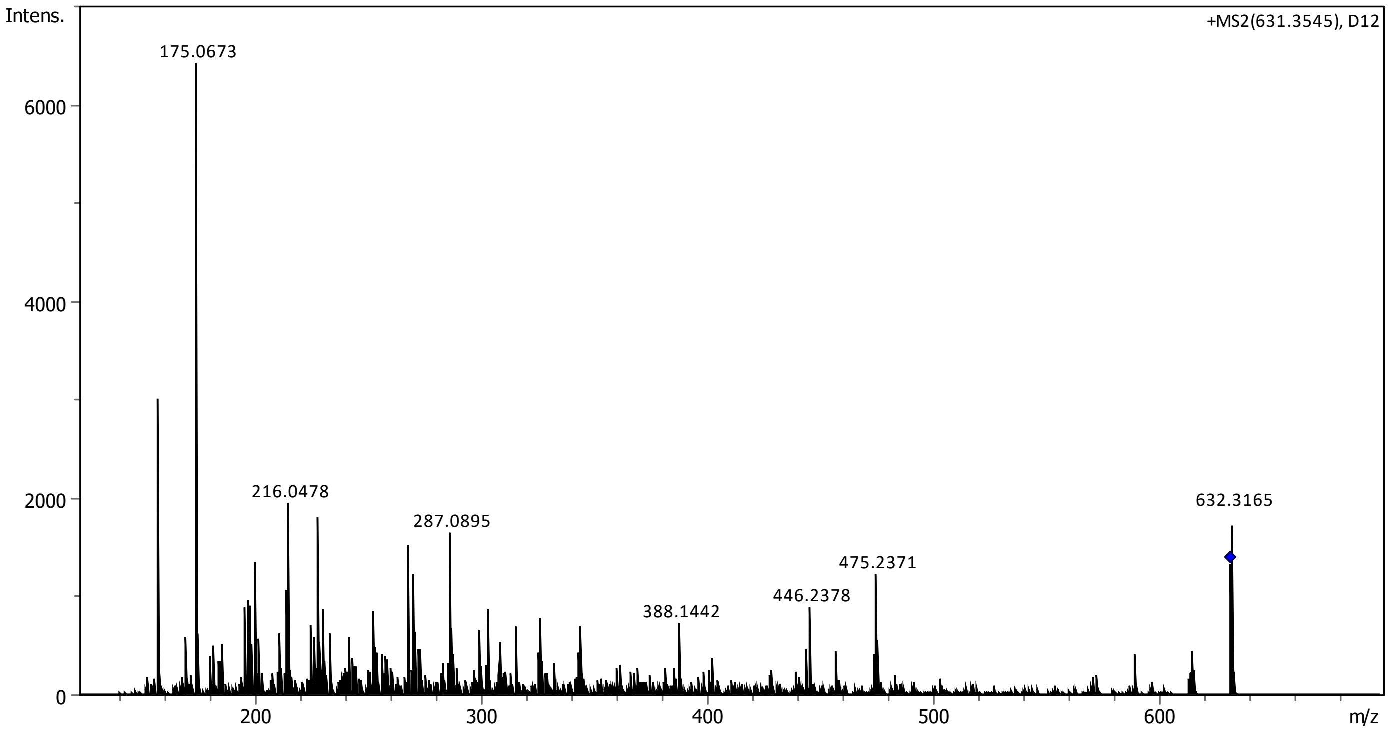


**Figure S4.** MS/MS fragmentation spectra utilized for the tentative identification of *m*/*z* = 632.3.

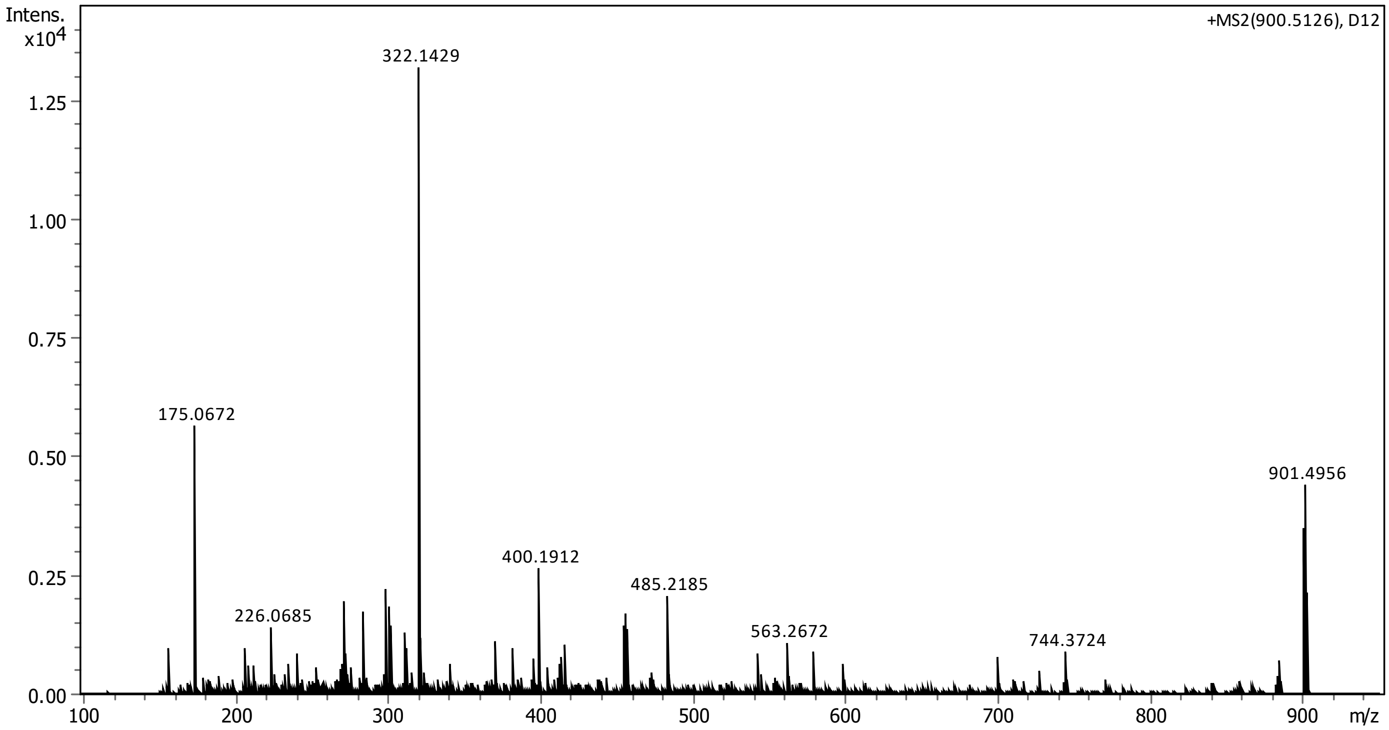


**Figure S5.** MS/MS fragmentation spectra utilized for the tentative identification of *m*/*z* = 900.5.

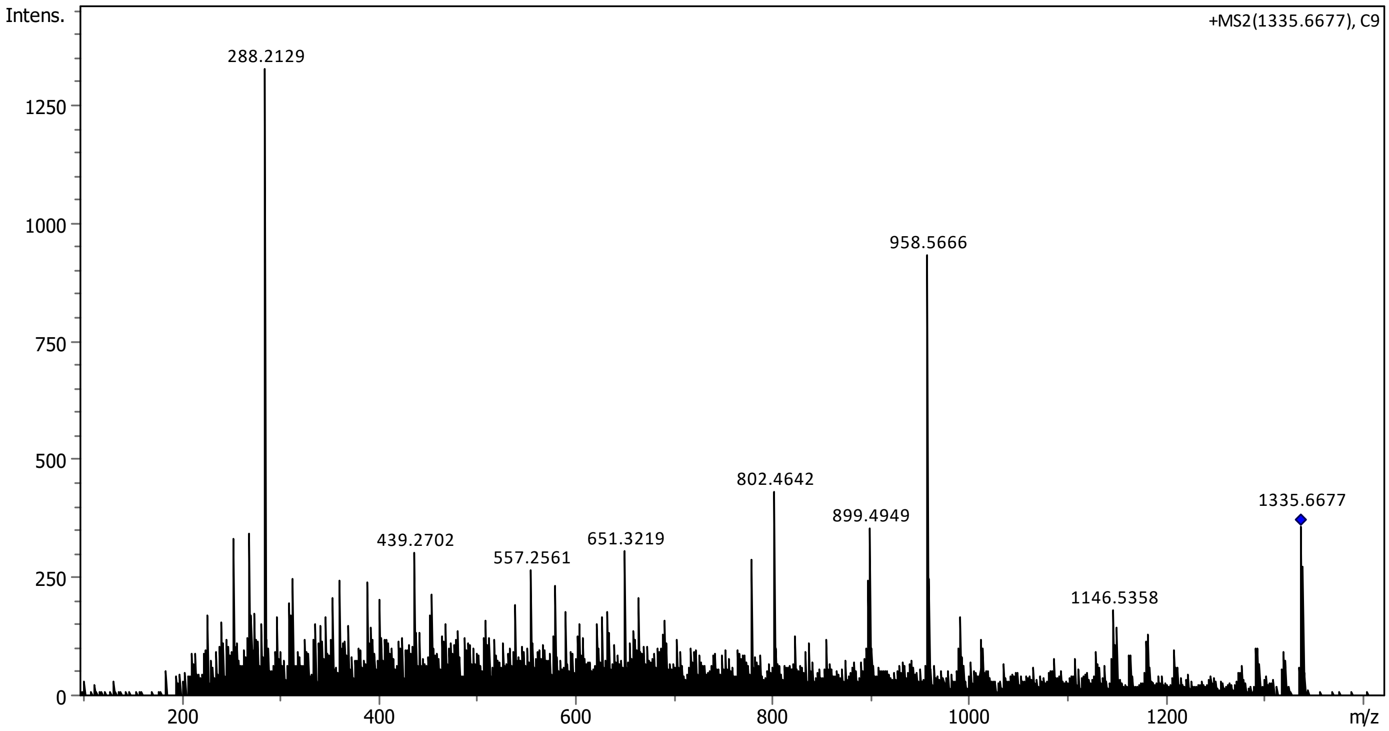


**Figure S6.** MS/MS fragmentation spectra utilized for the tentative identification of $m/z$ = 1335.7.

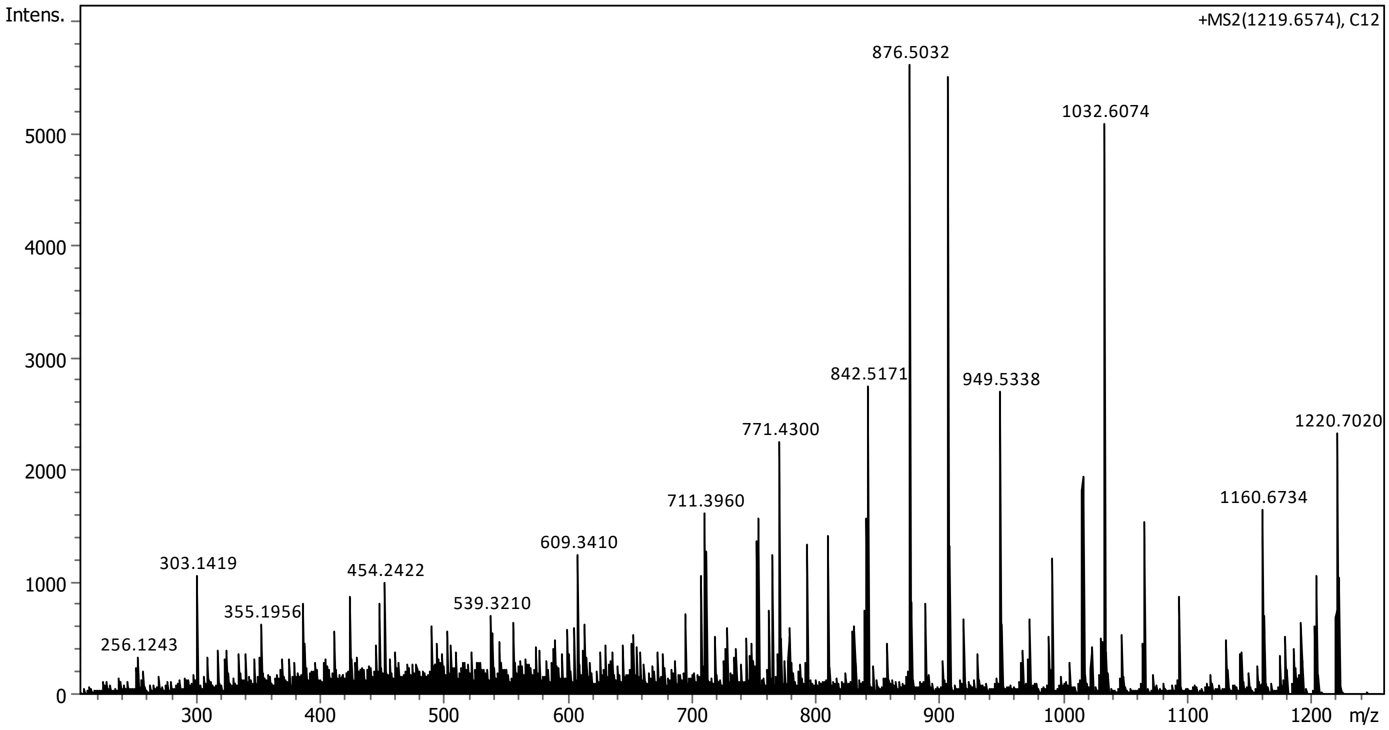


**Figure S7.** MS/MS fragmentation spectra utilized for the tentative identification of $m/z$ = 1220.7.

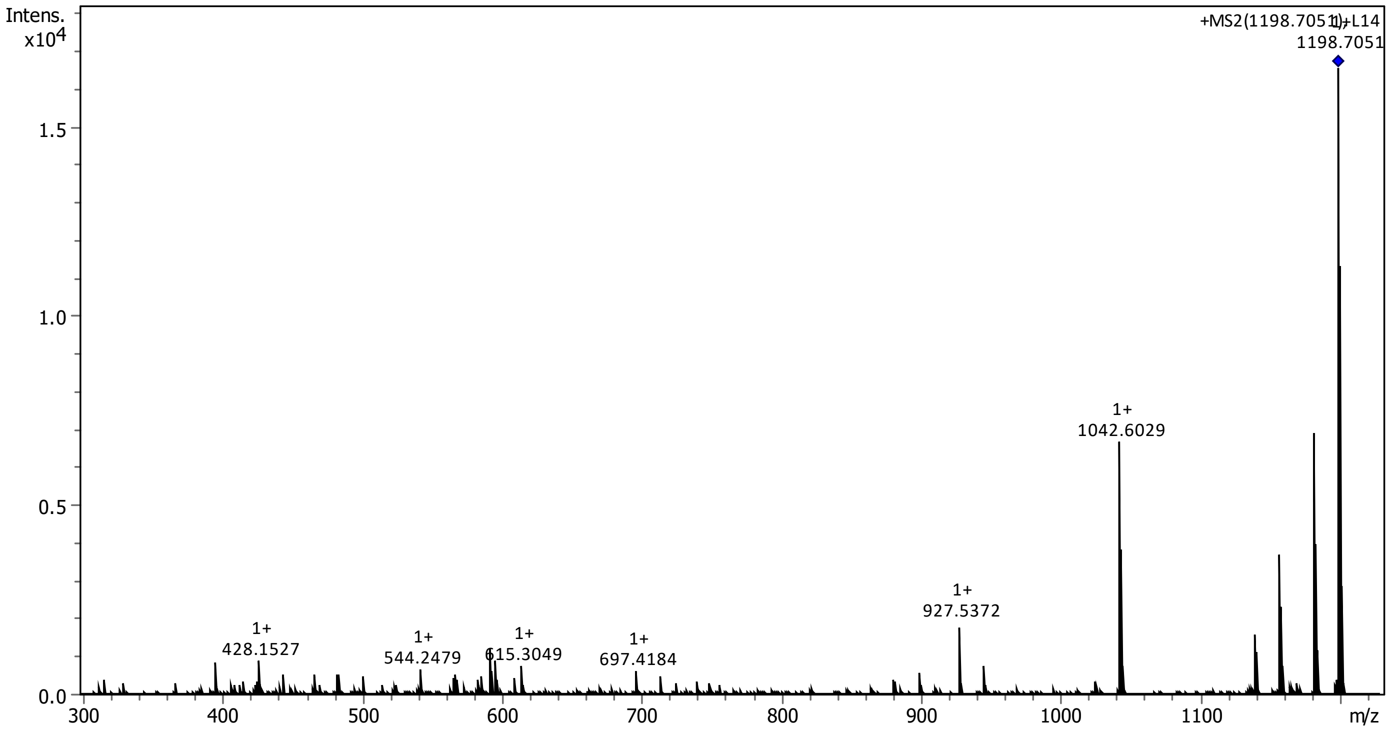


**Figure S8.** MS/MS fragmentation spectra utilized for the tentative identification of *m*/*z* = 1198.7.

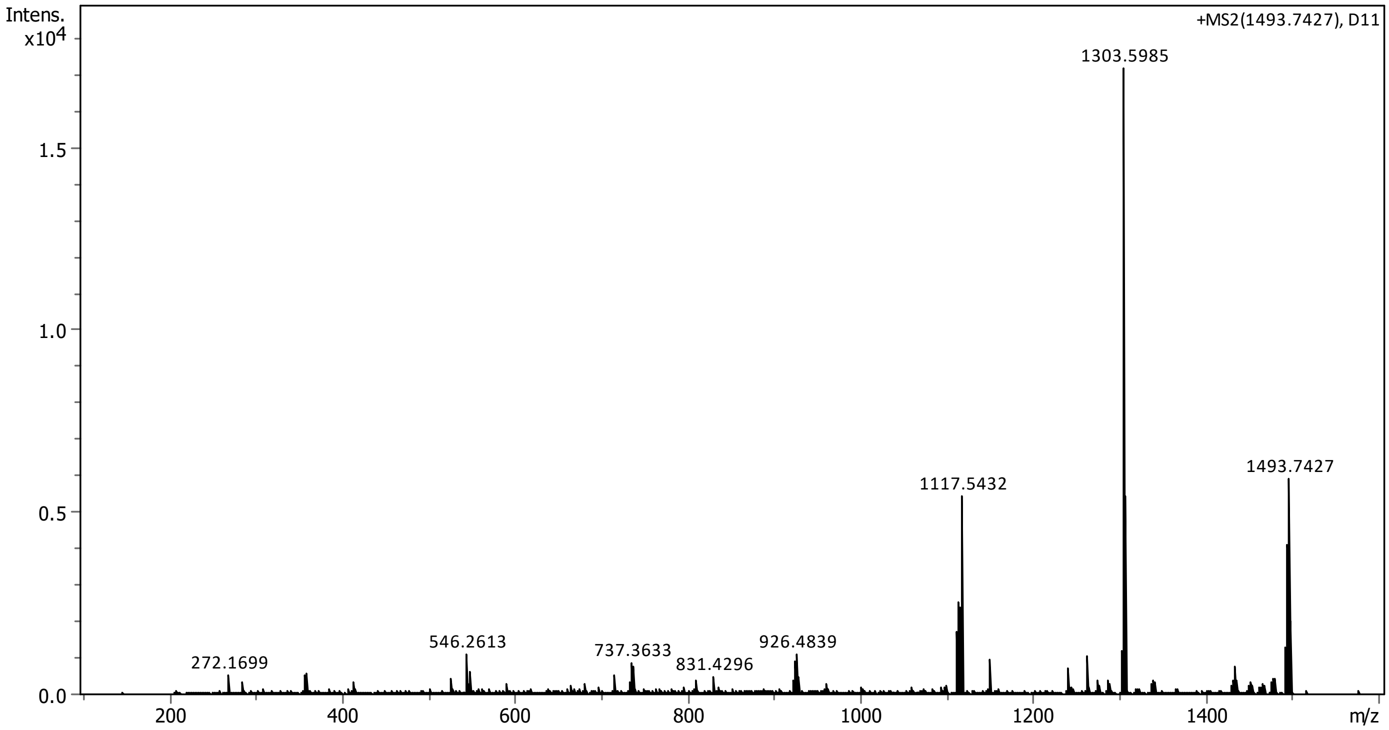


**Figure S9.** MS/MS fragmentation spectra utilized for the tentative identification of *m*/*z* = 1493.7.
